# Preparation and Physicochemical Characterization of Softgels Cross-Linked with Cactus Mucilage Extracted from Cladodes of *Opuntia Ficus-Indica*

**DOI:** 10.3390/molecules24142531

**Published:** 2019-07-11

**Authors:** Luis R. Camelo Caballero, Andrea Wilches-Torres, Agobardo Cárdenas-Chaparro, Jovanny A. Gómez Castaño, María Carolina Otálora

**Affiliations:** 1 Grupo Química-Física Molecular y Modelamiento Computacional (QUIMOL), Facultad de Ciencias, Universidad Pedagógica y Tecnológica de Colombia (UPTC), Avenida Central del Norte, Tunja 150001, Boyacá, Colombia; 2 Grupo de Investigación en Ciencias Básicas (NÚCLEO), Facultad de Ciencias e Ingeniería, Universidad de Boyacá, Tunja 150001, Boyacá, Colombia

**Keywords:** gelatin, cactus mucilage, softgels, crosslinking, nutritional

## Abstract

A new crosslinking formulation using gelatin (G) and cactus mucilage (CM) biopolymers was developed, physicochemically characterized and proposed as an alternative wall material to traditional gelatin capsules (softgels). The effect of G concentration at different G/CM ratios (3:1, 1:1 and 1:3) was analyzed. Transparency, moisture content (MC), solubility in water (SW), morphology (scanning electron microscopy, SEM), vibrational characterization (Fourier transform infrared, FTIR), color parameters (CIELab) and thermal (differential scanning calorimetry/thermogravimetric analysis, DSC/TGA) properties of the prepared composite (CMC) capsules were estimated and compared with control (CC) capsules containing only G and glycerol. In addition, the dietary fiber (DF) content was also evaluated. Our results showed that the transparency of composite samples decreased gradually with the presence of CM, the G/CM ratio of 3:1 being suitable to form the softgels. The addition of CM decreased the MC, the SW and the lightness of the capsules. Furthermore, the presence of polysaccharide had significant effects on the morphology and thermal behavior of CMC in contrast to CC. FTIR spectra confirmed the CMC formation by crosslinking between CM and G biopolymers. The addition of CM to the softgels formulation influenced the DF content. Our findings support the feasibility of developing softgels using a formulation of CM and G as wall material with nutritional properties.

## 1. Introduction

Soft gelatin capsules, now better known as softgels, have become one of the most specialized delivery systems used in the pharmaceutical and food industries for the safe supply of dietary nutrients, cosmetics and medicines to humans and animals. Softgels allow the reliable supply of liquid and semisolid dosages of bioactive compounds, due to the excellent hermeticity of their walls—which avoids the contact of their contents with light and oxygen—and their easy dissolution in biological fluids at body temperature [1]. Therefore, they are widely used as an oral administration form of drugs and vitamins, as well as suppositories (rectal or vaginal) for medicine dosage. Other uses include topical and ophthalmic preparations and the like.

Traditionally, the softgel structure is manufactured from animal-derived gelatin (G), water (30%), non-volatile plasticizer(s) such as glycerol and sorbitol (20–30%), and other minor additives, such as opacifiers and colorants [2,3]. The main source of G is collagen from the skin and bone of animals that is thermally denatured or partially degraded by acid, alkaline or enzymatic hydrolysis [4]. Production of G implies the pretreatment of the animal material, as well as the hydrolysis, extraction and recovery procedures that take several weeks, and differences in such processes have great effects on the properties of the final G products. Gelatin type A (isoelectric point at pH 9.0, extracted mainly from pork skin) and gelatin type B (isoelectric point at pH ranging from 4.7 to 5.3, manufactured from animal bones) are the main raw materials used in softgel manufacturing. In addition to the high costs of production of G, other drawbacks of softgels have been pointed out, like low consumer acceptability due to their animal-derived ingredients, physical and chemical instability, especially under moist conditions, and the release mechanisms of the encapsulated contents [3]. Thus, new trends in the food and pharmaceutical industry focus on the study of new capsules (soft and hard) composed of non-gelatin polymers, where the gelatin is partially or totally replaced, and which exhibit specific functionalities. 

Among the possible natural products for the total or partial substitution of G derived from animals in soft capsules, biopolymers extracted from plants, such as mucilage, gum and starch, have become a very attractive alternative. For instance, Hernández-Nava recently attained the complex coacervation between type B gelatin (GE) and chia mucilage (ChM) as an alternative to encapsulating agents [5]. On the other hand, da Silva reported the formation of polyelectrolyte complexes (PECs) based on type-A gelatin and gum Arabic [6], finding that the optimal complexation pH is the key parameter to obtain reproducible microcapsules with targeted features, regardless of the origin of the gum. Likewise, Tanner et al. [7], studied starch and its derivatives in combination with other polymers as gelatin substitutes in the production of soft capsules, using iota-carrageenan as plasticizer. 

In particular, cactus mucilage (CM) extracted from cladodes of *Opuntia ficus-indica* is an interesting natural hydrocolloid substitute for the partial replacement of G in the formation of softgels. It presents a molar mass of 3 × 10^6^ gmol^−1^ and is composed of L-arabinose (24.6–42.0%), d-galactose (21.0–40.1%), d-xylose (22.0–22.2%), l-rhamnose (7.0–13.1%) and α-d-(1→4) galacturonic acid (8.0–12.7%), as well as insoluble and soluble fiber [8,9,10,11,12,13,14]. This water-soluble hetero-polysaccharide is considered a functional biopolymer due to its beneficial health properties, such as anti-ulcer, anti-inflammatory, cytoprotective and cholesterol-lowering activities, for which it is widely applied in the pharmaceutical industry [14,15,16,17,18]. In addition, CM is utilized in the nano- and micro-encapsulation of bioactive compounds and as a natural super-plasticizer in the food industry [13,19,20,21].

To date, some studies have been published on the potential use of CM to form films [22,23,24,25]. However, to our knowledge, no study has been reported on the development of soft capsules based on CM with beneficial to health (mucilage–dietary fiber) and nutritional (gelatin–protein) properties. This study is aimed at developing soft capsules (softgels) built using G/CM mixtures, and at making a comparative evaluation of their physicochemical, microstructural and thermal properties with those of traditional soft capsules manufactured with gelatin (control sample). In addition, the total dietary fiber content was assessed.

## 2. Results and Discussion

### 2.1. Physicochemical Properties

#### 2.1.1. Transparency

**Figure 1:**
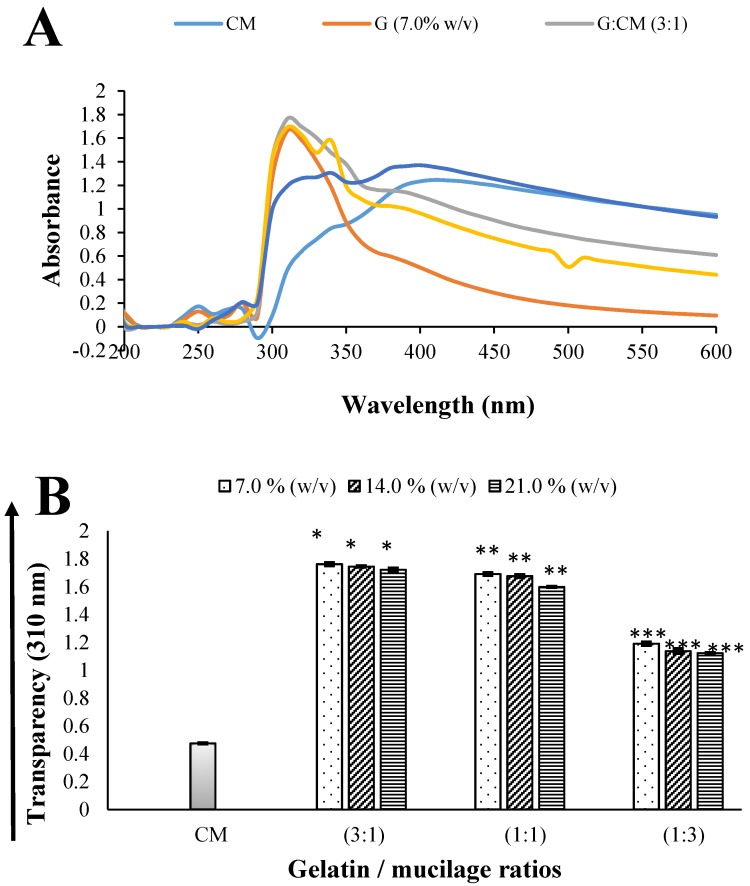
Ultraviolet (UV)-visible absorbance spectra (**A**) and transparency at 310 nm (**B**) of soft capsule-forming solutions as a function of concentration of Gelatin (G) and of the gelatin/cactus mucilage (G/CM) ratios (3:1, 1:1 and 1:3). All data are the mean of measurements (n = 3) ± standard deviation. Different asterisks in the same column for each solution indicate a statistical difference (*p* < 0.05) between samples.

Figure 1B shows the visible absorbance at 310 nm for G/CM solutions at ratios of 3:1, 1:1 and 1:3, each as a function of concentration of G (7.0%, 14.0% and 21.0% *w*/*v*), along with the corresponding absorbance of the stock solutions of G and CM. The G solution was relatively clear, while the CM solution showed an opaque appearance (Figure 1A). The increase in the concentration of G from 7 to 21% caused a non-significant (*p* > 0.05) and inversely-proportional decrease in transparency, independent of the G/CM ratio used. The above can be correlated with the fact that at higher concentrations of G (21.0% *w*/*v*), a greater crosslinking effect between CM and G is expected, thus forming a more compact and, therefore, less translucent network. However, both the presence and the increase of CM in the solutions significantly decreased (*p* < 0.05) the transparency of the samples; see Figure 1. It is also noted that the transparency of the CM stock solution was lower than that of all solutions of different ratios, indicating that the opacity of the solutions is attributed to the crosslinking of CM with G [26], which is in line with our Fourier transform infrared (FTIR) analysis (see Section 2.2.2.).

The transparency values in the G/CM (1:3) solutions were decreased significantly with respect to the G/CM (3:1) solutions; a behavior that could be attributed to the increase in the intermolecular interactions and therefore a reduced mobility of the biopolymer chains [27]. Additionally, the G/CM (1:3) composite samples showed a loss of gelation capacity at room temperature due to an increase in the proportion of CM in the mixture. The greater proportion of CM restricts the ability of the gelatin molecules to form triple-helix chains; hence, a less rigid network is obtained, with a consequent decrease in crosslinking density [28]. Therefore, concentration G (21.0% *w*/*v*), in a G/CM ratio of 3:1, was chosen to form composite capsules (CMC), which were physicochemically, structurally and thermally characterized.

#### 2.1.2. Moisture Content

The moisture contents of CMC and control capsules (CC) were 16.09% ± 2.18% and 21.20% ± 0.47%, respectively. The addition of CM to the composite capsule affects the water uptake (*p* < 0.05), in contrast to CC; this is probably due to the formation of hydrogen bonds between CM and G biomolecules that block the hydroxyl positions of the polysaccharides capable of associating with water [29].

#### 2.1.3. Solubility in Water

The solubilities in water of CMC and CC were 44.94% ± 2.61% and 51.56% ± 2.90%, respectively. Differences were found (*p* < 0.05) by the addition of CM to the matrix. The solubility value of the composite capsule was higher than that reported by Jamróz et al. [30] and Liu et al. [31]; this is probably due to a decrease in the inter-polymeric complexation between G and CM biopolymers that reduce the formation of intermolecular networks. The CCs showed a greater solubility in water than that reported by Oladzadabbasabadi et al. [32], probably due to the degree of ionization of gelatin [33].

The presence of CM in the composite capsule decreased slightly the solubility in comparison to CC. CM might interrupt the intermolecular association between polymeric chains, thus favoring a reduction in the porosity space and allowing water molecules to penetrate and dissolve polymeric moieties of the sample more easily, resulting in deformation of the sample [34]. Such a reduction in water solubility could then be attributed to a strong interaction between G and CM, as will be highlighted by our FTIR measurements. Similar results were reported by Wang et al. [35], who showed that the addition of polysaccharide was also able to decrease the solubility in water of a corn starch–collagen composite film. In particular, the formation of composite capsules with a reduction in water solubility is desirable for the delivery of nutrients, functional foods and pharmaceutical ingredients in gastro-intestinal environments, due to prolongation of the disintegration time of the matrix.

#### 2.1.4. Color Parameters

The color parameters (L*, a*, b* and ΔE*) of the CMC and CC are shown in Table 1. The incorporation of CM decreased significantly (*p* < 0.05) the lightness (L*) value of the soft capsule, most probably because of the dark color of the CM itself, thus affecting the clarity of the sample [36]. The ΔE* of CC and CMC increased significantly (*p* < 0.05) from 12.25 ± 0.87 to 25.45 ± 1.79, respectively, due to the presence of mucilage in the composite capsule, leading to more blurred capsule [37].

### 2.2. Structural Properties

#### 2.2.1. Scanning Electron Microscopy (SEM)

**Figure 2:**
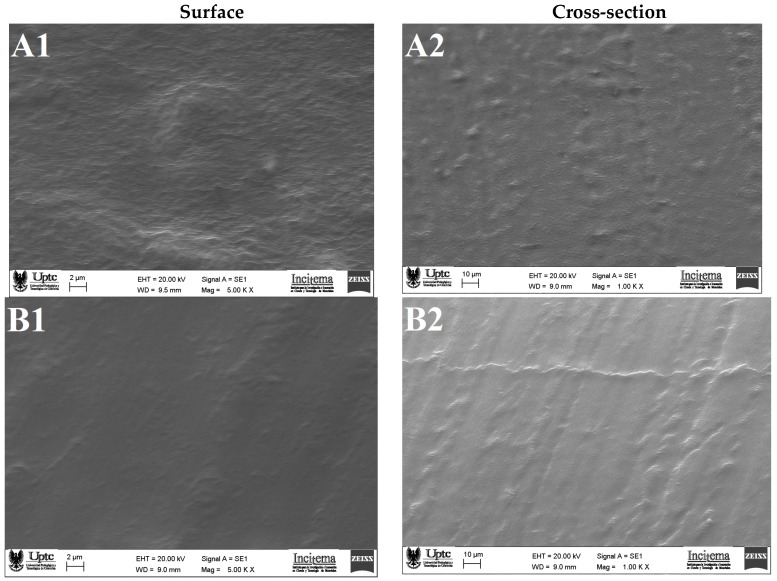
Representative micrograph images of capsule surfaces (5.00 KX) and cross-section areas (1.00 KX): (**A**) composite capsule and (**B**) control capsule.

The scanning electron microscope (SEM) micrographs of CMC and CC are presented in Figure 2. The composite capsule (Figure 2A1) revealed a rough and bumpy surface. A morphology like this suggests interaction between G and CM, which is in line with our FTIR analysis (see Section 2.2.2) and also indicates the presence of insoluble material (mucilage) in the capsule-forming solution [27]. Jamróz et al. [30], observed similar results in furcellaran–gelatin films. The micrographics of the cross-section (Figure 2A2) show short, scattered craters that indicate some heterogeneity in the matrix, which may have influenced the high water solubility (see Section 2.1.3).

The CC (Figure 2B1) exhibited a regular and relatively smooth surface. However, the cross-sectional images (Figure 2B2) presented rough microstructures with the presence of protuberances, which might be due to the shrinkages of matrix during the drying process.

#### 2.2.2. Fourier Transform Infrared (FTIR) Spectroscopy

**Figure 3:**
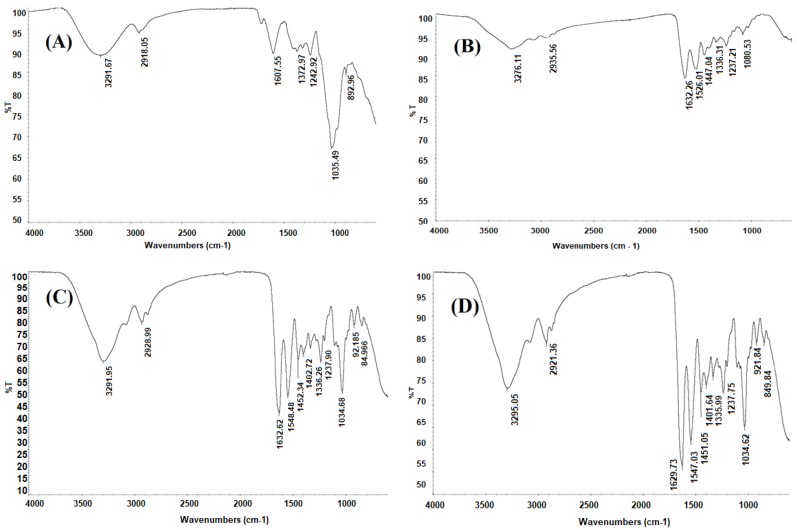
Fourier transform infrared (FTIR) spectra: (**A**) CM, (**B**) gelatin, (**C**) control capsule and (**D**) composite capsule, scanned at wavenumbers 4000–400 cm^−1^.

The FTIR spectrum of CM (Figure 3A) shows features very similar to those reported in the review by Nharingo and Moyo [38] for the mucilage of cactus cladodes. The band around 3292 cm^–1^ is mainly related to stretching bands of the hydroxyl groups, –OH, of the carboxylic acids, amino acids, alcohols and water molecules. The bands with peak positions around 2918 cm^–1^ are assigned to CH_3_ stretching. Spectral features of the C=C ring are observed at 1608 cm^–1^. The bands observed at 1373, 1243 and 1036 cm^–1^ are attributed to the characteristic vibrational modes of the pyranose ring [39], and the band at 893 cm^–1^ is related to aromatic groups [38].

The FTIR spectrum of G is presented in Figure 3B. The band around 3276 cm^–1^ is related mainly to the stretching vibration of the intramolecular hydrogen bond of the amino group of amide A, while the band observed around 2936 cm^–1^ is assigned to the C–H stretching of amide B [40]. The signal observed at 1632 cm^–1^ is assigned to C=O stretching of amide I, while the band around 1526 cm^–1^ is related to the N–H bending coupled with the C–N stretching of amide II. The peak at 1447 cm^–1^ is assigned to the C–N and N–H vibrations of amide III. Signals ranging between 1336 and 1080 cm^–1^ are associated to the symmetric and asymmetric deformation vibrations, respectively, of the methyl group [41].

The FTIR spectrum of CC is presented in Figure 3C. Compared to the FTIR spectrum of G (Figure 3B) the bands around 3276 and 2935.56 cm^–1^ are more intense and shifted to lower frequencies; this is possibly due to the –OH group contribution made by the plasticizer [42]. The intense band at 1630 cm^–1^ is due to C=O. Both C=O and N–H bonds easily form intermolecular hydrogen bonds with the O–H of glycerol. The band around 1035 cm^–1^ is attributed mainly to the intermolecular interactions between glycerol (C–O stretching) and gelatin [31].

The FTIR spectrum of CMC is presented in Figure 3D compared to control capsule (CC—Figure 3C) revealed in general slight variations in the intensity and amplitude of the peaks. For instance, the bands at 2921, 1630, 1547 and 1451 cm^−1^ in CC appeared red-shifted compared to those of CMC at 2928.99; 1632.62; 1548.48 and 1452.34 cm^−1^, which is clearly evidence of the formation of intermolecular interactions between functional groups of CM with G, thus indicating crosslinking between these polymers [26]. This is in line with the fact that the mucilage is a high molecular weight polysaccharide with large quantity of –OH groups and a relatively small amount of -COOH groups, which easily facilitates hydrogen bonding interactions with the –NH- groups in gelatin [36]. Luo et al. [36] have observed similar results in films formulated with G and chia mucilage.

### 2.3. Thermal Properties

**Figure 4:**
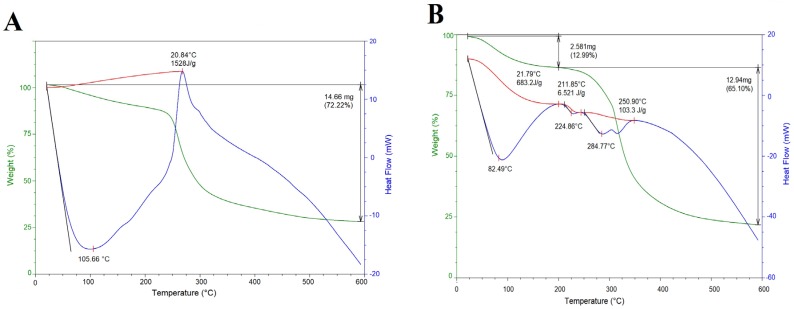

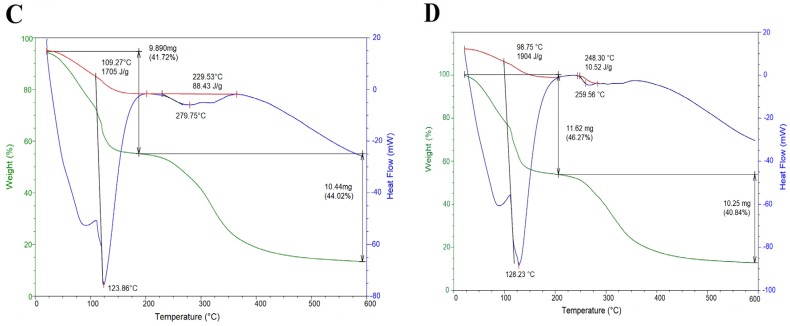
Representative DSC/TGA curves: (**A**) cactus mucilage (CM), (**B**) gelatin, (**C**) composite capsule and (**D**) control capsule.

Thermal analyses (differential scanning calorimetry/thermogravimetric analysis, DSC/TGA) of CM, G, CMC and CC are shown in Figure 4. The thermogram of CM (Figure 4A) showed a mass loss of 12.5% in the interval 25–225 °C (peak 105.66 °C), that corresponds to the loss of water. Successive thermal and gelatinization processes are correlated with a mass loss of 59.7% at 273 °C; this is attributed to degradation of the polysaccharide structure and decomposition/volatilization of material, respectively [13]. Similar thermal behavior has been reported for CM extracted from Opuntia spp. [43].

The thermogram of the second thermal event for the gelatin powder (Figure 4B) showed a mass loss of 12.9% in the range 25–200 °C (peak 82.49 °C) that is attributed to the melting temperature, evaporation of free and bound water [44]; followed by a mass loss of 65.1% at 224 °C attributed to the helix–coil transition temperature and to the protein thermal decomposition process. These values are similar to those reported for commercial gelatin [45].

The thermogram for the composite capsule (Figure 4C) presented two endothermic events. The first thermal event presented a mass loss of 41.7% in the interval of 25–175 °C (peak 123.86 °C), being higher than that reported for the individual mucilage and gelatin (Figure 4A,B), as result of the evaporation of moisture from the matrix. The second thermal event showed a mass loss of 44.0% at 279.75 °C, possibly from the evaporation of glycerol used as plasticizer for capsule formation [22] and degradation of gelatin and mucilage. The decomposition temperature of the capsule increased, in contrast to those of the individual polysaccharide (273 °C) and protein (224 °C). Thus, the addition of CM had an effect on the thermal stability of the capsule, stimulating interactions between the matrix components [29].

The thermogram of the CC (Figure 4D) exhibited two thermal events. The first event presented a mass loss of 46.2% in the range of 25–210 °C (peak 128.23 °C), being higher than that reported for the gelatin powder (82.49 °C) used in the design of the capsule. This finding demonstrated the greater thermal stability of CC than gelatin powder.

The above results showed an increase in thermal stability of the composite capsule (279.75 °C) in relation to the CC (259.56 °C), which can be a direct consequence of the crosslinking reaction between CM–G and Gly. This thermal behavior was similar to those reported by Nur Hazirah et al. [26] and Mujtaba et al. [22]. The high thermal stability of the composite capsule compared to control indicate that G/CM could preferably be used as shell material to encapsulate thermally sensitive bioactive compounds.

### 2.4. Dietary Fiber Content

The total dietary fiber content (TDF) was measured for the CM and CMC. The value for CM was about 72.1 g/100 g dry matter. This value was higher than those reported by Rodríguez-González et al. [14] (57.23 g/100 g dry matter) and Otálora et al. [13] (57.70 g/100 g dry matter) for *Opuntia ficus-indica* cladode mucilage. This difference in fiber content could be correlated to variation in cultivars and growing conditions. The value for CMC was about <4.0 g/100 g dry matter. This result reveals the contribution of polysaccharides in TDF content of the soft capsule, thus suggesting that the TDF content of the matrix can be controlled by the content of mucilage in the formulation of capsule. The incorporation of this dietary fiber represents a promising strategy for the development of soft capsules with beneficial to health properties.

### 2.5. Photograph of Composite Capsules (CMC) and Control Capsules (CC)

**Figure 5:**
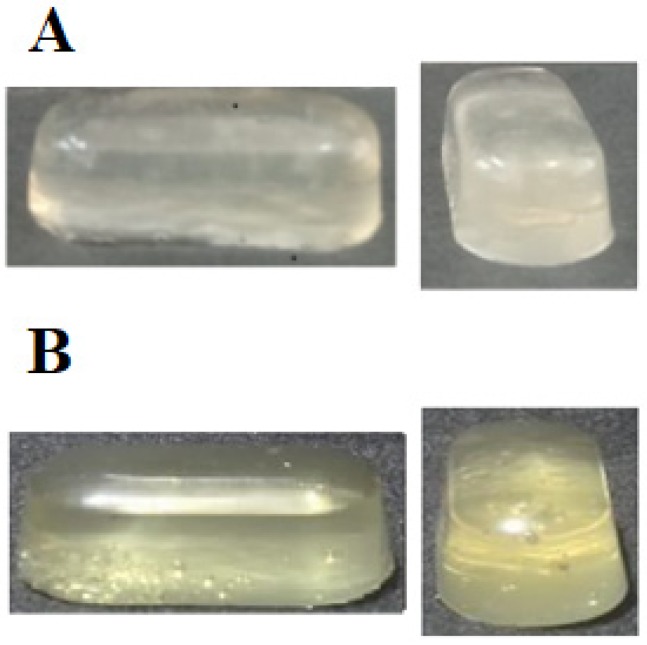
The appearance of soft capsules: (**A**) composite capsule and (**B**) control capsule.

The appearance of CMC and CC is shown in Figure 5. The composite capsule had a grainy and opaque appearance in contrast to the control. This is possibly due to the concentration and incomplete solubilization of the CM in the formulation of the CMC. The size of CMC and CC capsules (length 20 mm, diameter 9 mm) was relatively proportional to the size and elliptical shape of the mold. This matrix size is similar to that reported by Oishi et al. [46].

## 3. Materials and Methods 

### 3.1. Materials and Reagent

The CM used in this study was extracted from cladodes of *Opuntia ficus-indica*, obtained from local farmers in the municipality of Duitama (Boyacá, Colombia), following the methodology reported by Quinzio, Corvalán, Lopez and Iturriaga [47] and used as obtained without further purification. Food grade G (Type B, Bloom 285, isoelectric point 4.2–6.5; Mw 40,000–50,000 Da and ∼99% purity) provided by Gelco (Medellin, Colombia) was used to form soft capsules. Glycerol (Gly) provided by Merck (Darmstadt, Germany) was added as a plasticizer.

### 3.2. Preparation of Solutions

G (7.0, 14.0 and 21.0 g) and CM (1.0 g) were dissolved separately in 100 mL of distilled water at 40 and 18 °C for 30 min and 2 h, respectively, under constant stirring at 300 rpm using magnetic stirring (C-MAG HS 7S000, IKA, USA). The G solutions (7.0, 14.0 and 21.0% *w*/*v*) were mixed separately with glycerol (Gly) at a concentration of 15% (*w*/*v*) at 60 °C for 2 h to remove residual air bubbles and to obtain a homogeneous solution. The G/Gly mixtures will be referred to as the control sample. Three different G/CM solutions, at relative ratios of 3:1, 1:1 and 1:3 *w*/*w*, were prepared for each G concentration of 7.0%, 14.0% and 21.0% *w*/*v*, by constant magnetic stirring for 1 h at room temperature. The CM/G/Gly mixtures will be referred to as composite samples. 

### 3.3. Formation of Soft Capsules

Each formulation (composite and control samples) was poured into a mold of elliptical shape (length 22 mm, diameter 11 mm) and dried in a vacuum oven (UM 400, Memmert, Schwabach, Germany) at 25 °C in a relative humidity of 40% for 1 h. These conditions correspond to those used industrially for the manufacture of soft capsules [48]. The composite and control capsules will be referred to as CMC and CC, respectively.

### 3.4. Physicochemical Properties

#### 3.4.1. Transparency Measurements of Soft Capsule-Forming Solutions

Transparency of the composite and control samples were determined by measuring the absorbance at 310 nm in a glass cuvette (1 cm path length), using a DR 5000™ UV-vis spectrometer (Hach Company, Loveland, CO, USA). Distilled water was used as blank for all solutions tested. All samples were measured in triplicate at least.

#### 3.4.2. Moisture Content

The moisture content of the CMC and CC were determined gravimetrically by drying in a vacuum oven (UM 400, Memmert, Schwabach, Germany) at 25 °C, in a relative humidity of 40% for 12 h until constant weight (Method 934.06) [49]. The capsules after drying were conditioned in bags of high-density polyethylene and stored in a desiccator to room temperature with a relative humidity of 30%. In these conditions, the equilibrium moisture content of the shell material and oxygen permeability through the material are minimal, what improving the stability of the capsule minimizing changes physicochemical [1].

#### 3.4.3. Solubility in Water

The method described by Wang et al. [35] was followed with some modifications. After drying (Section 3.4.2), the weighed capsules were immersed in 30 mL of distilled water and maintained under these conditions at 20 °C for 5 h. Afterwards, the capsules were taken out of the solution and dried in an oven at 25 °C until constant weight. The solubility in water of the capsules was calculated by using Equation (1):

(1) $$\mathrm{Solubility}\;\left(\%\right)=\frac{\left(\mathrm{Initial}\;\mathrm{dried}\;\mathrm{weight}\;\mathrm{of}\;\mathrm{soft}\;\mathrm{capsules}-\mathrm{Final}\;\mathrm{dried}\;\mathrm{weight}\;\mathrm{of}\;\mathrm{soft}\;\mathrm{capsules}\right)\;}{\mathrm{Initial}\;\mathrm{dried}\;\mathrm{weight}\;\mathrm{of}\;\mathrm{soft}\;\mathrm{capsules}}\times 100$$

#### 3.4.4. Color Parameters

The CIE*Lab* parameters (L*, a*, b*) of CMC and CC were measured using a Minolta CR-400 colorimeter (Osaka, Japan). The instrument was calibrated using a white standard with color coordinates of L* (lightness/brightness) standard = 94.98, a*(redness/greenness) standard = −0.54 and b* (yellowness/blueness) standard = 3.59, provided by Minolta. The total color difference (ΔE*) was calculated using Equation (2):

(2) $$\begin{array}{l} \Delta E^{*}=[{\left(L\times \mathrm{capsule}-L\times \mathrm{standard}\right)}^{2}+{\left(a\times \mathrm{capsule}-a\times \mathrm{standard}\right)}^{2}\\ +{\left(b\times \mathrm{capsule}-b\times \mathrm{standard}\right)}^{2}]\times 0.5 \end{array}$$

### 3.5. Structural Properties

#### 3.5.1. Scanning Electron Microscopy (SEM)

Morphology of the surface and cross-section of CMC and CC were evaluated by scanning electron microscope (EVO MA 10, Carl Zeiss, Oberkochen, Germany), operating at 20 kV, coating the samples with gold–palladium sputtering before examination.

#### 3.5.2. Fourier Transform Infrared (FTIR) Spectroscopy

FTIR spectra of CM, G, CMC and CC were recorded on a Thermo Scientific Nicolet iS10 spectrometer (Waltham, MA, USA) equipped with an attenuated total reflection (ATR) accessory. The spectra were collected over the region 4000–400 cm^−1^ at a resolution of 4 cm^−1^.

### 3.6. Thermal Properties

Thermogravimetric analysis (TGA)/differential scanning calorimetry (DSC) was performed on a TA Instrument (SDT Q600 V20.9 Build 20, New Castle, USA). Argon was used as a purge gas (100 mL/min). The samples of CM, G, CMC and CC were placed in alumina pans and heated from 20 to 600 °C at a heating rate of 10 °C/min.

### 3.7. Dietary Fiber Content

Soluble and insoluble dietary fiber content were determined in CM and CMC according to the AOAC 991.43 enzymatic-gravimetric method [49].

### 3.8. Statistical Analysis

The results were reported as mean ± standard deviation (n = 3). The Kruskal–Wallis test was performed to identify differences among the means using InfoStat/P version1.1 statistical software [50]. Differences at probability level *p* ˂ 0.05 were considered significant.

## 4. Conclusions

The present study contributes in the promotion of cladode mucilage as an alternative source of raw material for the manufacture of soft capsules with a reduced content of animal-derived gelatin, which constitutes a current topic of interest for the food and pharmaceutical industries. The gelatin/mucilage combination has been successfully used as capsule material. The addition of mucilage, a natural hydrocolloid rich in dietary fiber, influenced the physicochemical, morphological and thermal characteristics compared to the traditional capsules manufactured using gelatin. Obtained results suggest that G/CM combination may be a potential coating material with nutritional value, in the delivery and release of bioactive compounds in the gastro-intestinal environment. 

## Figures and Tables

**Table 1:** Experimental CIELab color parameters (L*, a* and b*), total color difference (*∆*E*) for composite and control capsules.

Parameter	Composite Capsule	Control Capsule
L*	71.38 ± 1.93 ^a^	87.73 ± 0.88 ^b^
a*	−1.80 ± 0.02 ^c^	−1.97 ± 0.08 ^c^
b*	13.07 ± 0.53 ^d^	13.36 ± 0.43 ^d^
ΔE*	25.45 ± 1.79 ^e^	12.25 ± 0.87 ^f^

All data are the mean of measurements (n = 3) ± standard deviation. Different letters in the same row and column for each color parameters indicate a statistical difference (*p* < 0.05) between samples with the presence and absence of mucilage.
